# Supplementary material for: Mechanistic studies of mycobacterial glycolipid biosynthesis by the mannosyltransferase PimE
Source: Nat Commun. 2025 Apr 29;16:3974. doi: 10.1038/s41467-025-57843-1 (PMC12041525; doi:10.1038/s41467-025-57843-1)
Supplement: Supplementary file 1 — Supplementary Information [file 41467_2025_57843_MOESM1_ESM.pdf]

Mechanistic studies of mycobacterial glycolipid biosynthesis by the mannosyltransferase PimE

Yaqi Liu[1], Chelsea M. Brown[2,3], Nuno Borges[4,5], Rodrigo N. Nobre[4], Satchal Erramilli[6], Meagan Belcher Dufrisne[1,7], Brian Kloss[1], Sabrina Giacometti[1,8], Ana M. Esteves[4], Cristina G. Timóteo[4], Piotr Tokarz[6], Rosemary J. Cater[1,9], Todd L. Lowary[10,11,12], Yasu S. Morita[13], Anthony A. Kossiakoff[6], Helena Santos[4,*], Phillip J. Stansfeld[2,*], Rie Nygaard[1,14,*] and Filippo Mancia[1,*]

[1]Department of Physiology and Cellular Biophysics, Columbia University Irving Medical Center, New York, NY, USA.

[2]School of Life Sciences and Department of Chemistry, University of Warwick, Coventry, UK.

[3]Groningen Biomolecular Sciences and Biotechnology Institute and Zernike Institute for Advanced Materials, University of Groningen, Nijenborgh, The Netherlands.

[4]Instituto de Tecnologia Química e Biológica António Xavier, ITQB NOVA, Universidade Nova de Lisboa, Oeiras, Portugal.

[5]Marine and Environmental Sciences Centre, Escola Superior de Tecnologia, Instituto Politécnico de Setúbal, Setúbal, Portugal.

[6]Department of Biochemistry and Molecular Biophysics, University of Chicago, Chicago, IL, USA.

[7]Department of Chemistry, University of Virginia, Charlottesville, VA, USA.

[8]School of Medicine, New York University, New York, NY, USA.

[9]Institute for Molecular Bioscience, The University of Queensland, St. Lucia, Queensland 4072, Australia.

[10]Institute of Biological Chemistry, Academia Sinica, Nangang, Taipei, Taiwan.

[10]Institute of Biological Chemistry, Academia Sinica, Nangang, Taipei, Taiwan.[11]Department of Chemistry, University of Alberta, Canada.

[12]Institute of Biochemical Sciences, National Taiwan University, Taipei, Taiwan.

[13]Department of Microbiology, University of Massachusetts, Amherst, MA, USA.

[14]Department of Radiation Oncology, Weill Cornell Medicine, New York, NY, USA.

[*]Correspondence to be addressed to: santos@itqb.unl.pt (H.S), Phillip.Stansfeld@warwick.ac.uk (P.S), rin7007@med.cornell.edu (R.N), fm123@cumc.columbia.edu (F.M.)

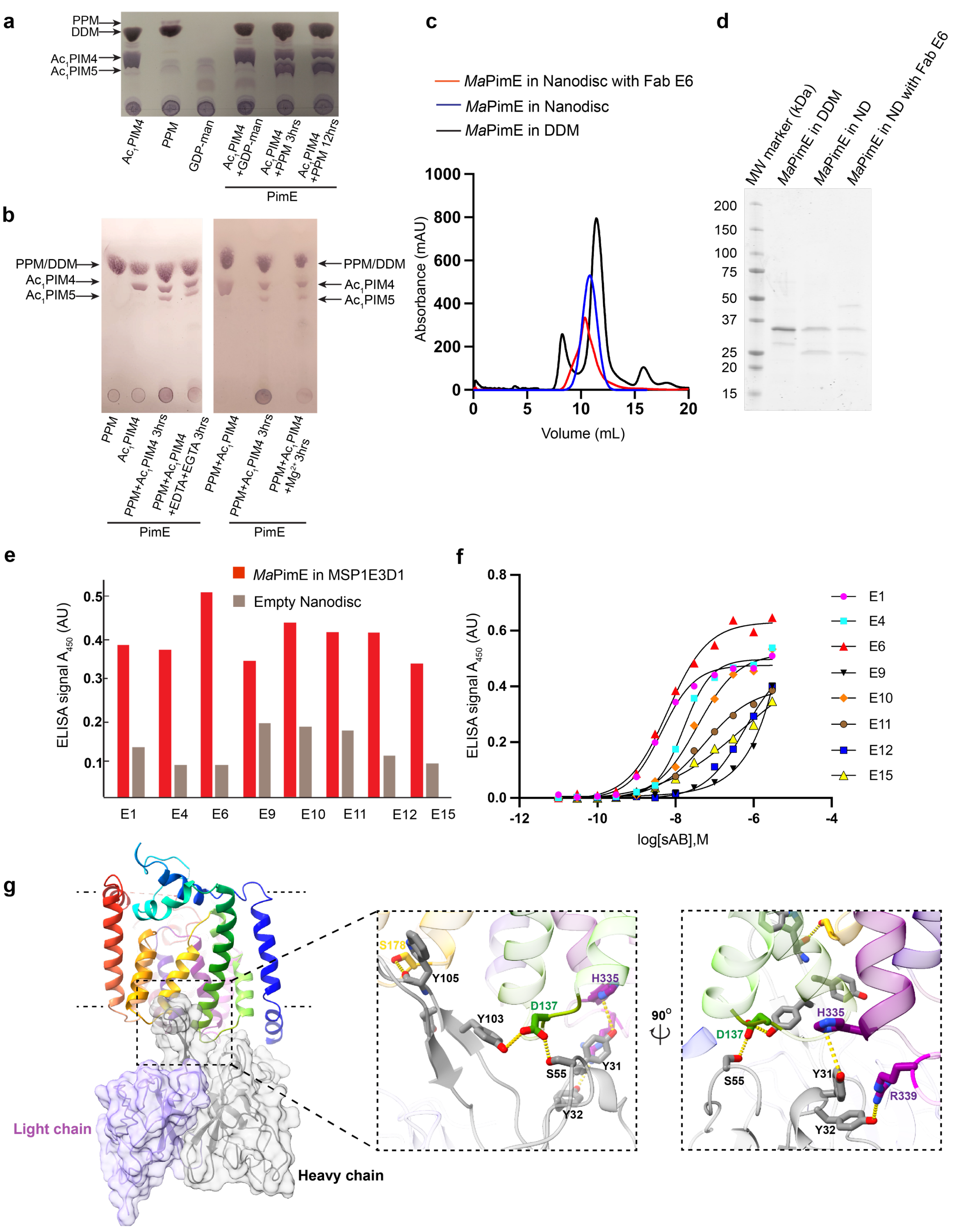

Supplementary Fig. 1 Purification and characterization of *Ma*PimE.

(a) TLC analysis of the enzymatic activity of *Ma*PimE in the membrane fraction, confirming the formation of Ac_1PIM5 from the substrates Ac_1PIM4 and PPM. Lanes (left to right): Ac_1PIM4 alone; PPM alone; GDP-mannose alone; PimE membrane fraction incubated with Ac_1PIM4 and PPM for 3 hours; PimE membrane fraction incubated with Ac_1PIM4 and PPM for 12 hours. Formation of Ac_1PIM5 product confirms successful mannosyl transfer. Glycolipids were visualized by orcinol staining. (b) Metal dependency analysis of *Ma*PimE. Left TLC plate - lanes (left to right): Membrane fraction with PPM only; Membrane fraction with Ac_1PIM4 only; Membrane fraction with both PPM and Ac_1PIM4; Membrane fraction with PPM, Ac_1PIM4, and metal chelators (EGTA and EDTA). The positions of PPM/DDM, Ac_1PIM4, and Ac_1PIM5 are indicated by arrows. Right TLC plate - lanes (left to right): mixture of Ac_1PIM4 and PPM; membrane fraction incubated with Ac_1PIM4 and PPM; membrane fraction incubated with Ac_1PIM4 and PPM supplemented with $MgCl_2$. The positions of PPM/DDM, Ac_1PIM4, and Ac_1PIM5 are indicated by arrows. (c) SEC elution profiles of purified *Ma*PimE in detergent (black), incorporated into a nanodisc (blue) and incorporated into a nanodisc with Fab-E6 bound (red). (d) SDS-PAGE gel of *Ma*PimE purification. The first lane shows *Ma*PimE purified in DDM, the second lane shows *Ma*PimE reconstituted into nanodiscs (using MSP1E3D1 and POPC), and the third lane shows *Ma*PimE reconstituted into nanodiscs (MSP1E3D1 and POPC) with Fab-E6 bound. (e) Single-point ELISA of Fab clones (E1, E4, E6, E9, E10, E11, E12, and E15) against *Ma*PimE, demonstrating the specific binding of the Fabs to *Ma*PimE compared to empty nanodiscs. Data are presented as a bar chart from a single experiment, where the bar height corresponds to the measured signal intensity for each Fab clone. (f) Multi-point ELISA of selected Fabs against *Ma*PimE, showing the concentration-dependent binding of the Fabs to *Ma*PimE in nanodiscs. Data points represent individual measurements from a single experiment. (g) The PimE and Fab-E6

binding interface, highlighting the key residues involved in the interaction between the Fab and the cytoplasmic loops of *Ma*PimE.

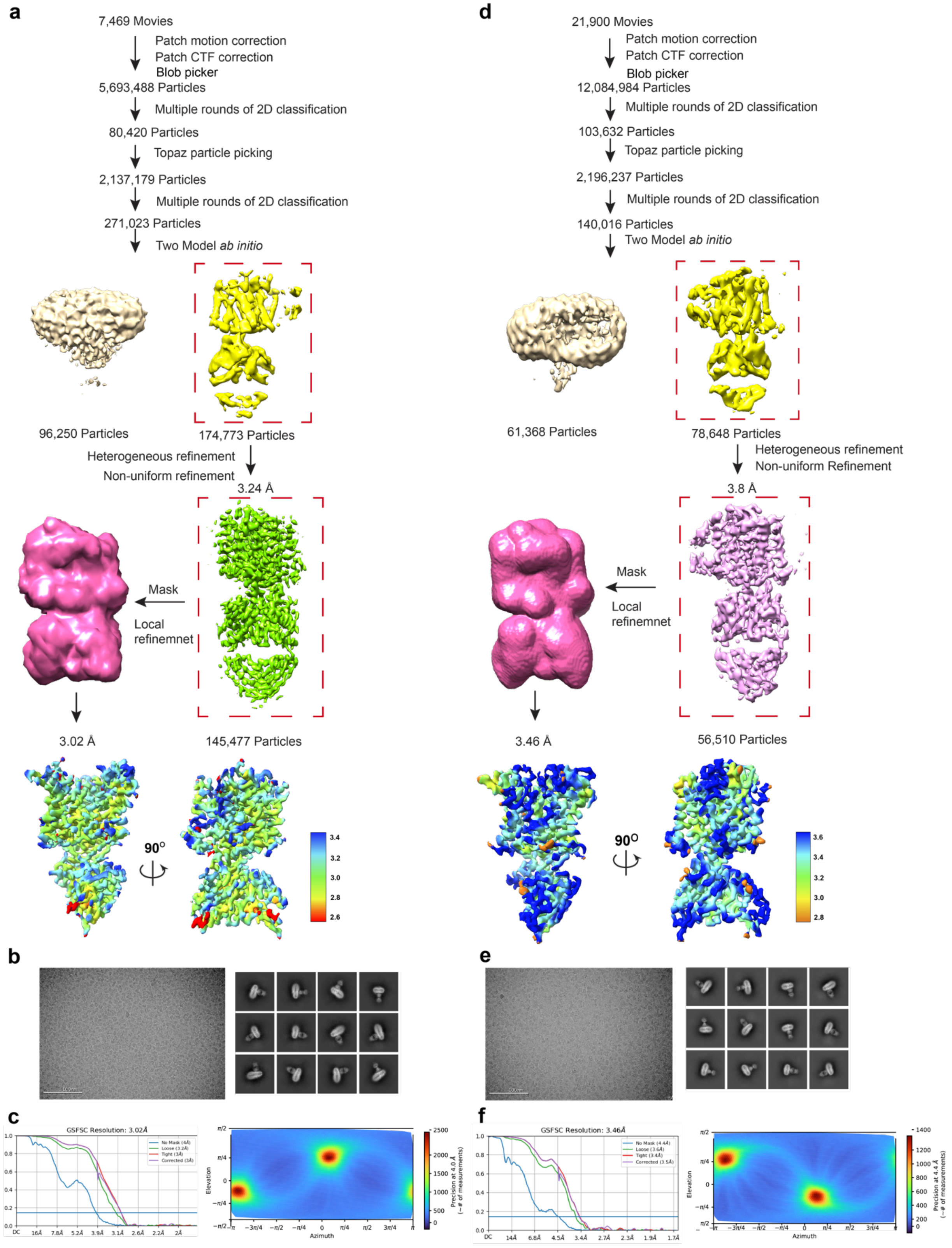

Supplementary Figure 2. Cryo-EM data processing and validation.

(a-c) Data processing workflow for the apo structure of *Ma*PimE, including particle picking, 2D classification, *ab initio* model generation, heterogeneous refinement, non-uniform refinement, and local refinement. The final map has a resolution of 3.02 Å (FSC = 0.143). (d-f) Data processing workflow for the substrate-bound structure of *Ma*PimE, following similar steps as in (a-c). The final map has a resolution of 3.46 Å (FSC = 0.143).

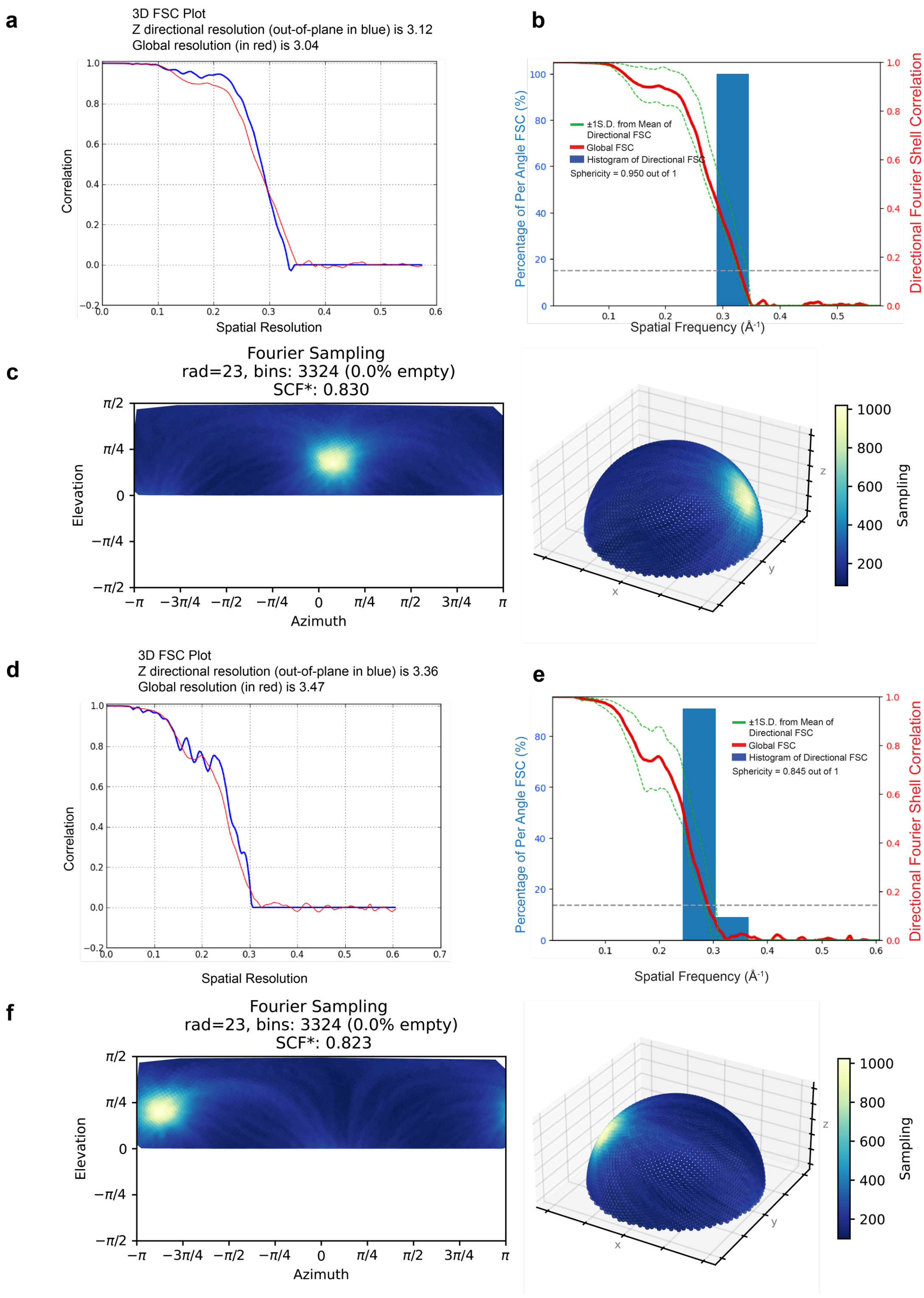

Supplementary Fig.3 Assessment of directional resolution and sampling quality for apo and product-bound *Ma*PimE reconstructions

(a-c) Analyses for apo *Ma*PimE reconstruction: (a) 3DFSC plot showing global directional resolution. The Z directional resolution (blue) is 2.89 Å, with a global resolution (red) of 2.90 Å. (b) Histogram of directional FSC values. The histogram quantifies variation in directional resolution, with the standard deviation (±1σ) shown, and a calculated sphericity value of 0.895, indicating isotropy of the protein density. (c) Sampling Compensation Factor (SCF) plot. The SCF value of 0.822 confirms sufficient sampling of Fourier space, exceeding the threshold of 0.81, which indicates adequate sampling distributions despite orientation bias[1]. (d-f) Analyses for product-bound *Ma*PimE reconstruction: (d) 3DFSC plot showing global directional resolution. The Z directional resolution (blue) is 3.31 Å, with a global resolution (red) of 3.30 Å. (e) Histogram of directional FSC values. The histogram quantifies directional resolution, with the standard deviation (±1σ) shown, and a sphericity value of 0.857, demonstrating isotropy of the protein density. (f) Sampling Compensation Factor (SCF) plot. The SCF value of 0.823 indicates robust sampling of Fourier space, confirming the adequacy of orientation distributions[1]. All 3DFSC plots and histograms were generated using 3DFSC software[2]. SCF plots were created using CryoSPARC[3] v4.4+.

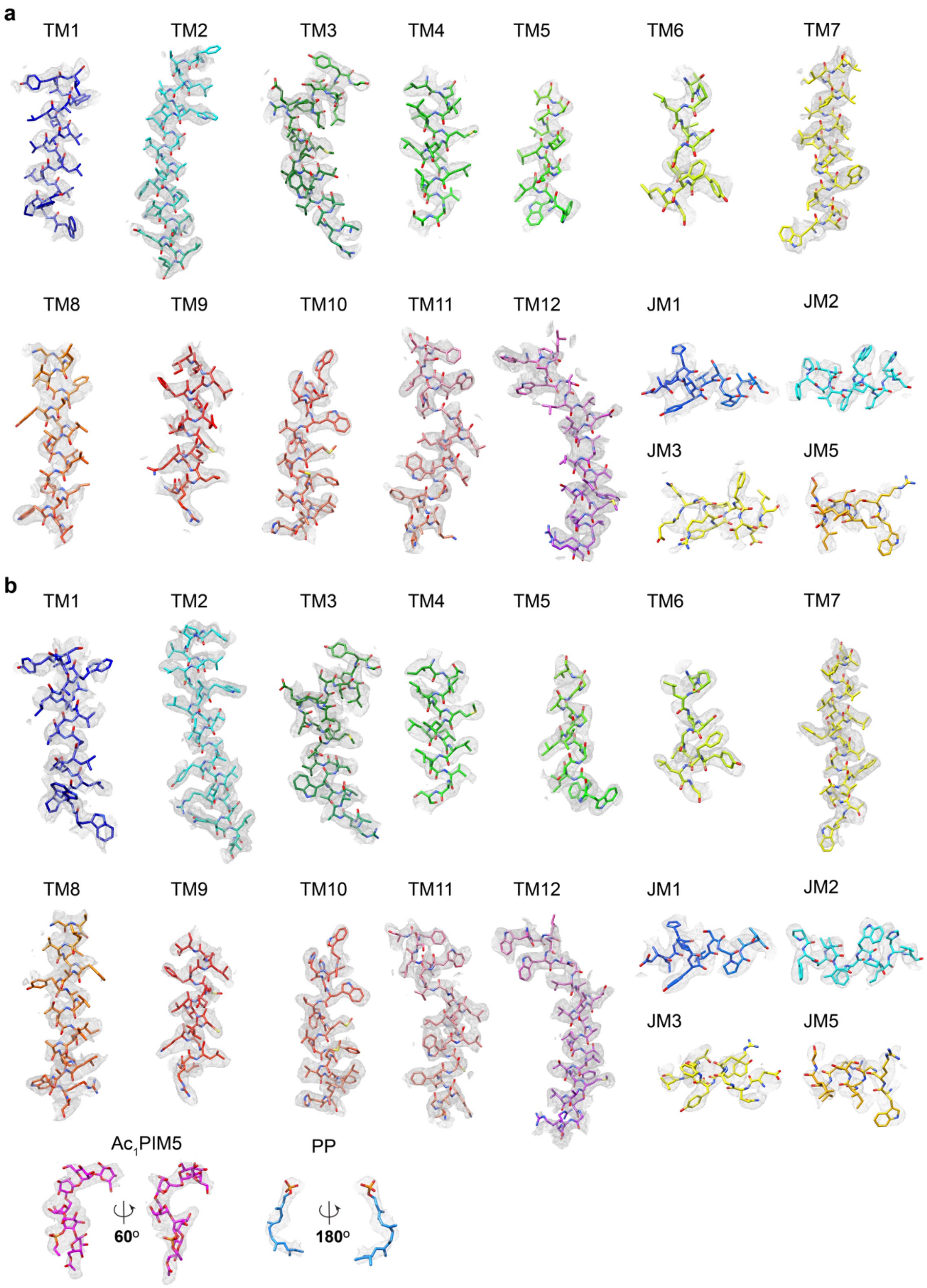

Supplementary Fig. 4 Cryo-EM density and model for the TM helices and juxtamembrane helices of apo and products-bound *Ma*PimE.

(a) cryo-EM density (mesh) and atomic model (stick representation) for transmembrane (TM) helices and juxtamembrane (JM) helices in apo *Ma*PimE. (b) cryo-EM density and atomic model for products-bound *Ma*PimE, shown as in (a).

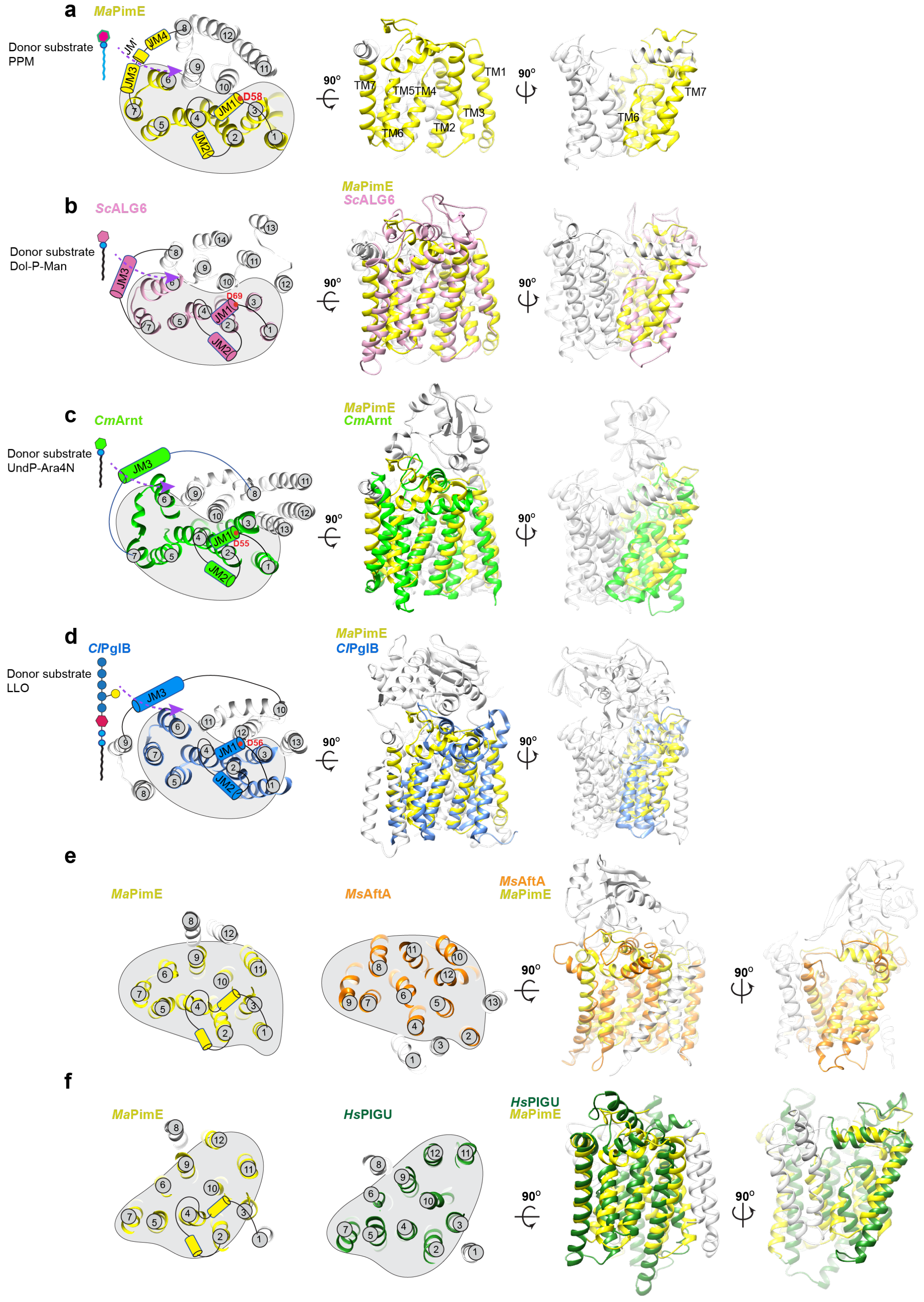

Supplementary Fig. 5 Comparison of PimE with structural homologs.

(a) The first seven TM helices of PimE are shown in yellow, while the remainder is shown in grey. (b-d) Superposition of PimE with ALG6 (b), ArnT (c), and PglB (d). The first seven TM helices of ALG6, ArnT, and PglB are colored in pink, yellow, and blue, respectively, while the remaining helices are shown in grey. The conserved catalytic residues (D58 in PimE, D69 in ALG6, D56 in ArnT, and D55 in PglB) are highlighted as sticks. The JM helices (JM1-JM3) and the conserved structural motif formed by the first seven TM helices are labeled. The donor substrates for each enzyme (PPM for PimE, Dol-P-Man for ALG6, UndP-Ara4N for ArnT, and LLO for PglB) are shown as cartoons, revealing a conserved mode of donor substrate binding among these GT-C glycosyltransferases. Z-scores from the DALI analysis for ALG6, ArnT, and PglB are 18.1, 9.2, and 6.5, respectively. (e) Superposition of PimE (yellow) with AftA (orange), a mycobacterial arabinosyltransferase (Z-score: 9.2). The helix bundle of PimE, containing TM helices 1-7 and 9-11, shows a similar architecture to the helix bundle of AftA, which contains TM helices 2 and 4-12. (f) Superposition of PimE (yellow) with PIGU (green), a subunit of the human GPI transamidase complex (Z-score: 19.7). The arrangement of the TM helices in PimE, particularly TM helices 2-7 and 9-12, shows a similar architecture to the arrangement of TM helices 2-7 and 9-12 in PIGU.

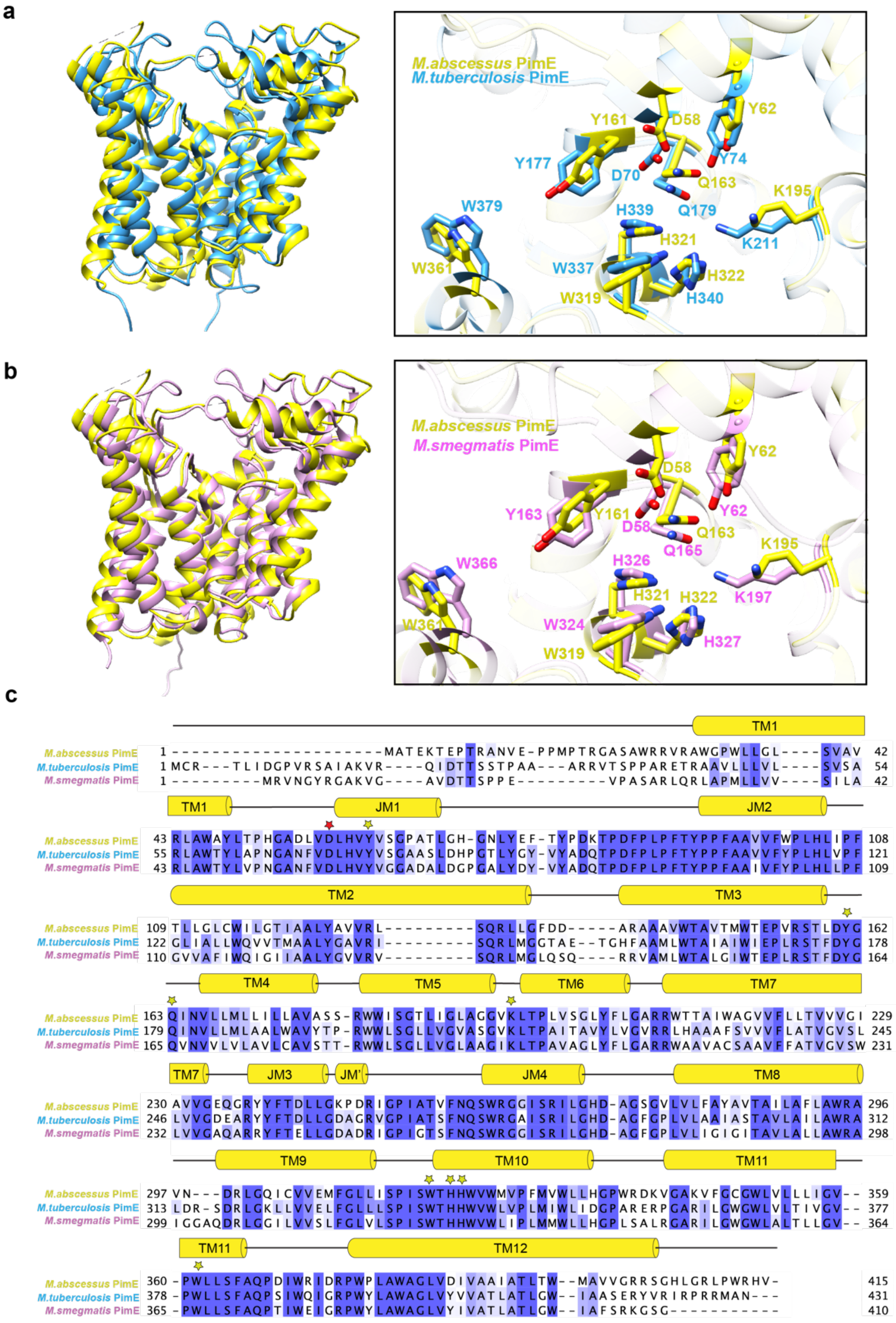

Supplementary Fig.6 Structural comparison of PimE from *M. abscessus*, *M. tuberculosis*, and *M. smegmatis*.

(a) Left: Structural superposition of the cryo-EM structure of *M. abscessus* PimE with AlphaFold-predicted models of *M. tuberculosis* PimE. Right: Magnified view of the active site region showing critical residues involved in catalysis and substrate coordination as stick representations. (b) Left: Structural superposition of the cryo-EM structure of *M. abscessus* PimE with AlphaFold-predicted models of *M. smegmatis* PimE. Right: Magnified view of the active site region showing critical residues involved in catalysis and substrate coordination as stick representations. (c) of PimE from the three mycobacterial species. *M. tuberculosis* PimE shares 64% sequence identity with M. *abscessus* PimE (RMSD of 2.498 Å), while *M. smegmatis* PimE shares 61% sequence identity (RMSD of 1.850 Å). The essential catalytic residue is marked with a red star, and other functionally important residues are indicated by yellow stars.

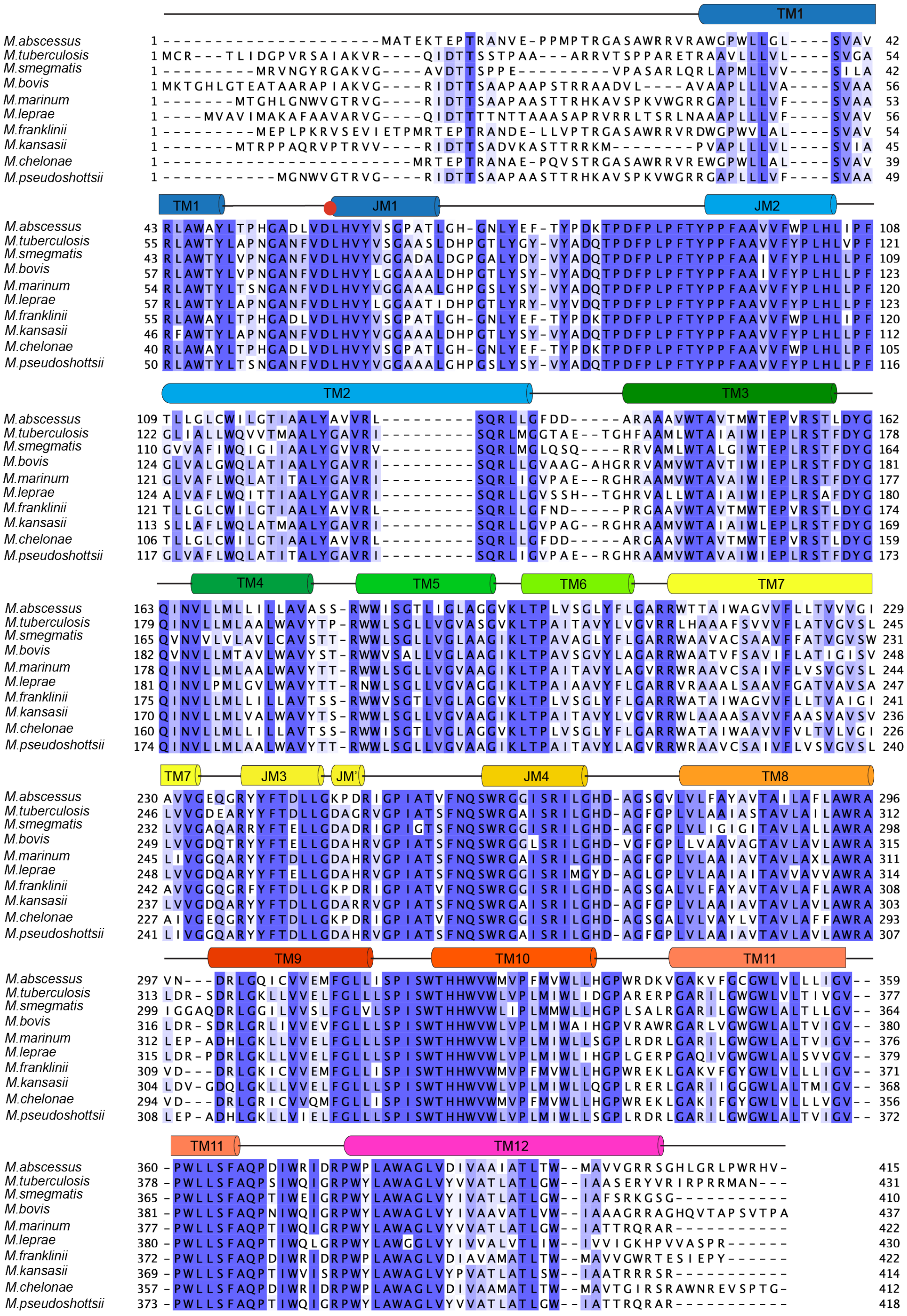

Supplementary Fig. 7 Sequence alignment of PimE orthologs from various mycobacterial species. The secondary structure elements (TM helices and JM helices) are indicated and the catalytically essential residue D58 is marked with red dot.

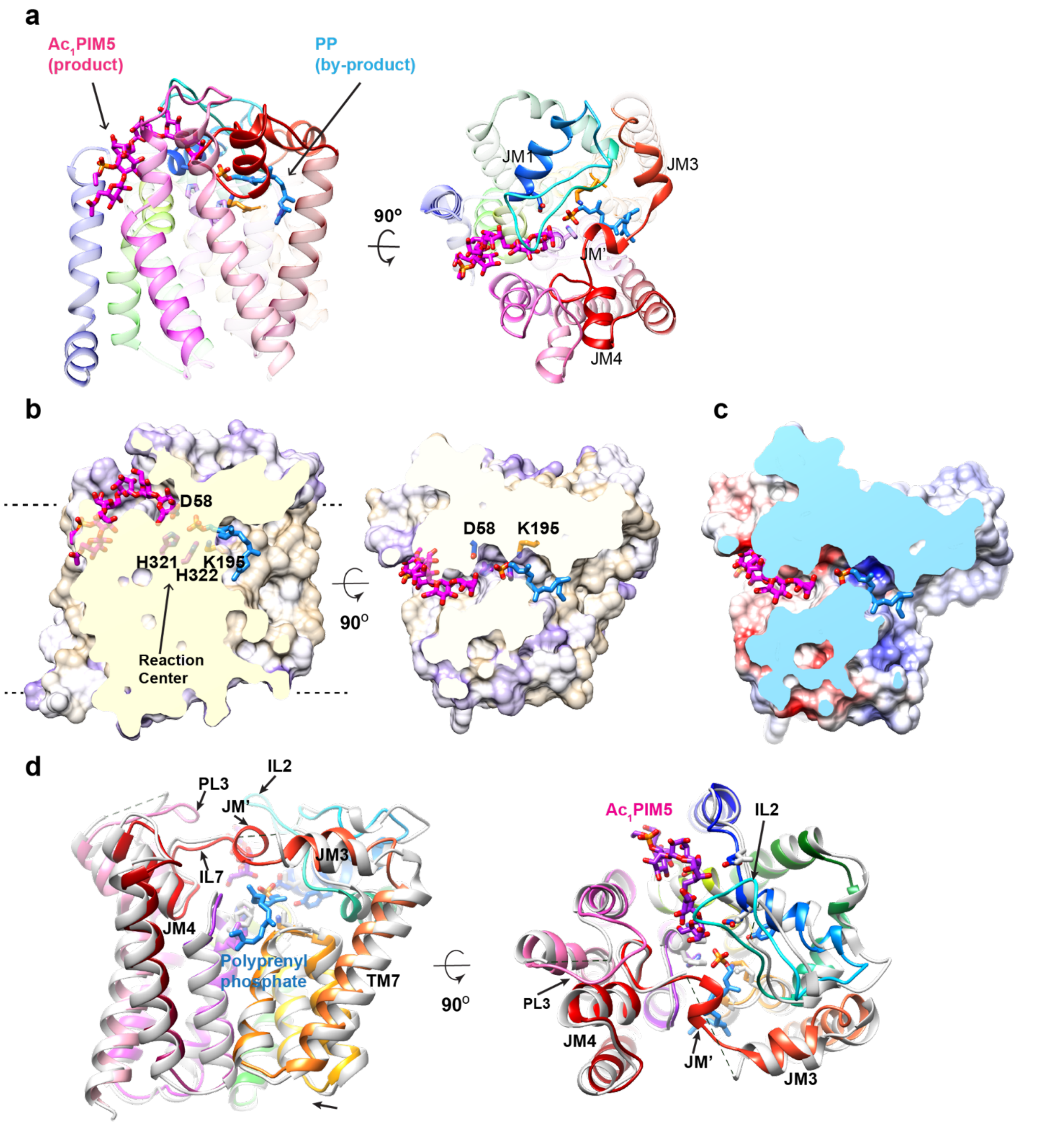

Supplementary Fig. 8 Product-bound complex of *Ma*PimE.

(a) Overview of the product-bound structure of *Ma*PimE, focusing on the positioning of PP and Ac_1PIM5 which are shown as sticks within PimE. (b) Hydrophobic surface representation of the

product-bound complex of *Ma*PimE, viewed parallel (left) and perpendicular (right) to the membrane plane. The reaction center, encompassing the conserved residues D58, K195, H321, and H322, is highlighted. The head groups of PP and Ac_1PIM5 converge at the reaction center, with the phosphate group of PP located close to K195 and the fifth mannose of Ac_1PIM5 positioned near D58. (c) Cross-section of the electrostatic surface of the product-bound complex of *Ma*PimE, viewed perpendicular to the membrane plane. Ac_1PIM5 is located along the negatively charged region of the cavity, while PP is situated along the positively charged region. (d) Superimposition of the product-bound *Ma*PimE structure (rainbow) and the apo *Ma*PimE structure (grey) highlighting the conformational rearrangements upon substrate binding, particularly in the periplasmic loops PL1, PL2, and PL3. The TM domain exhibits minimal deviations, with a modest inward pivot rotation/translation of TM helix 7 being the most notable change.

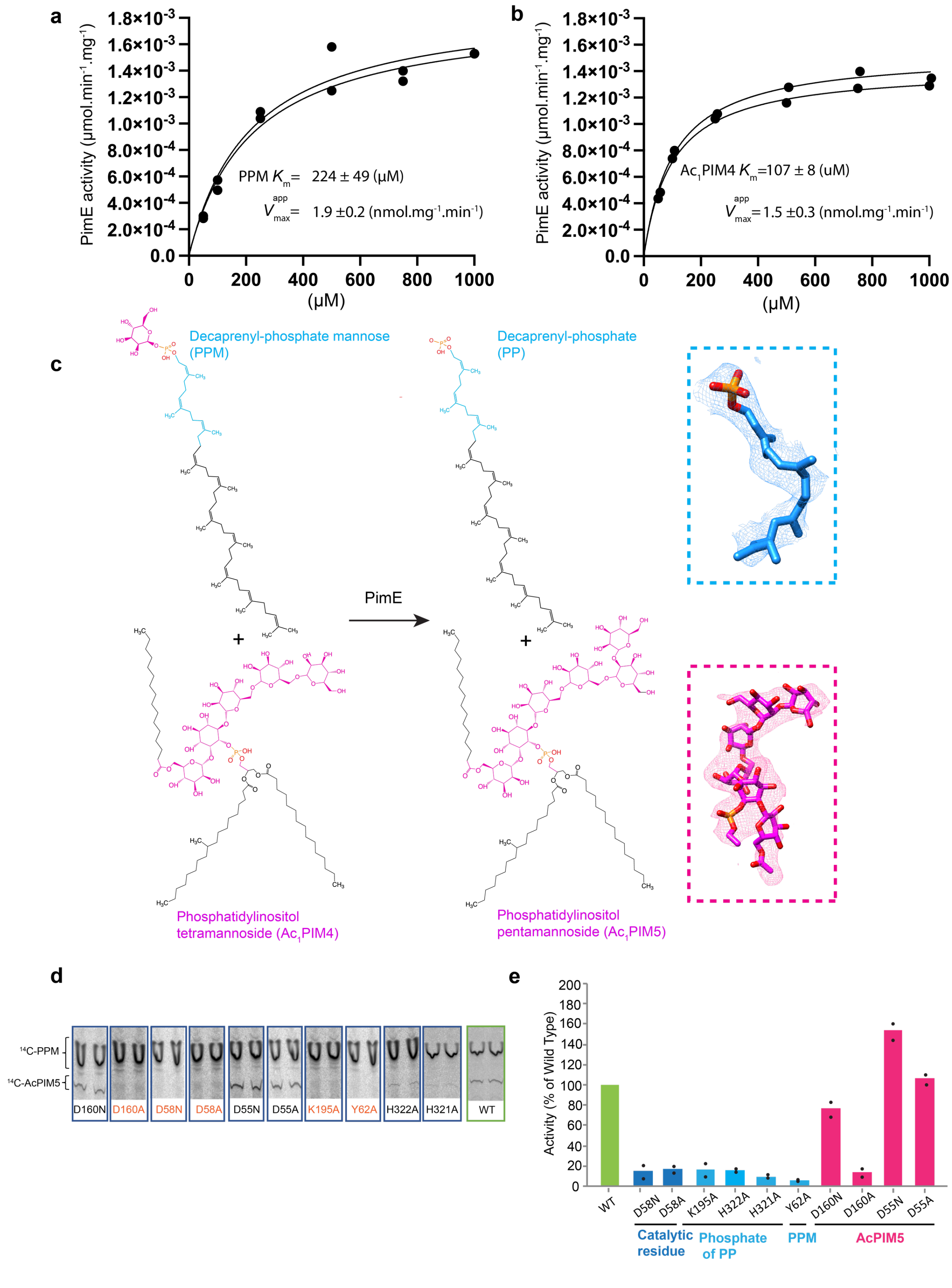

Supplementary Fig. 9 Kinetic characterization of *Ma*PimE.

(a-b) Michaelis-Menten plots for PPM (a) and Ac_1PIM4 (b), displaying the kinetic parameters (K_m and V_{max}) of *Ma*PimE for each substrate. Data points represent individual measurements from two independent experiments, with the solid line indicating the best-fit curve according to the Michaelis-Menten equation. The solid line indicates the best-fit curve according to the Michaelis-Menten equation. The K_m and V_{max} values, along with their standard deviations, are provided for each substrate. (c) Schematic representation of the enzymatic reaction catalyzed by PimE, showing the conversion of Ac_1PIM4 and PPM to Ac_1PIM5 and PP. The resolvable density for the carbon chain of PP is colored in blue, while the phosphate group is depicted with heteroatoms (phosphate in orange and oxygen in red). For Ac_1PIM5, the resolvable density is colored in magenta, with heteroatoms for oxygen (red) and phosphate (orange). (d) TLC analysis of the enzymatic activity of WT and mutant PimE using ^{14}C-labeled substrates. Mutating D58 to alanine or asparagine abolishes activity, while mutations of K195, H321, H322, and Y62 lead to reduced activity. Mutating D55 does not significantly affect activity, while mutating D160 to alanine largely reduces activity, but mutating it to asparagine only partially reduces activity. (e) Quantification of relative enzymatic activity of wild-type (WT) and mutant *Ma*PimE variants expressed in *E. coli*. The activity of WT *Ma*PimE was set to 100%, and the activities of mutant variants are expressed as percentages relative to WT. Bars represent the mean enzymatic activity of two independent experiments (n=2), with individual experimental values shown as dots. Mutations are grouped according to functional roles: catalytic residue (D58N, D58A), phosphate of PP binding (K195A, H322A, H321A), predicted PPM binding site (Y62A), and AcPIM5 binding (D160N, D160A, D55N, D55A).

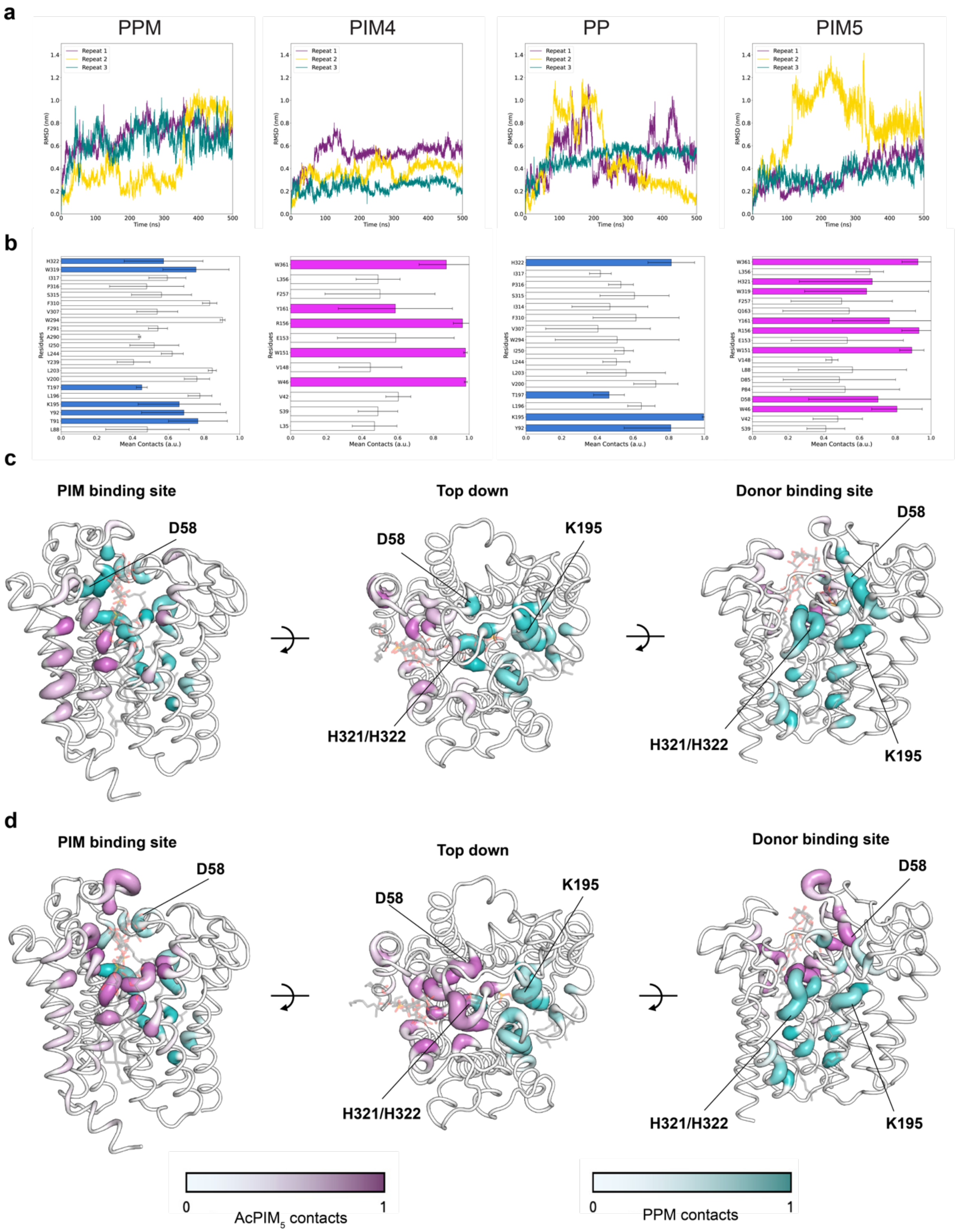

Supplementary Fig. 10 Molecular dynamics simulations of PimE interactions with substrates and products.

(a) The root mean squared deviation (RMSD) of the ligands simulated with PimE. The substrates (left) and products (right) are shown, with a line representing an independent repeat. (b) Contact graphs between PimE and ligands over the simulation lengths. The average of three simulations is shown, with the error bar representing standard error. Residues that we subjected to mutation studies are highlighted. A contact value of 1 represents contact with the ligand for the entire simulation; residues with contact values below 0.4 have been omitted for clarity. (c) Areas of PimE in contact with simulated ligands in simulations. The residues in contact with Ac_1PIM4 (purple) and PPM (blue). The darker the color, the more contacts throughout all simulations. The cartoon is thicker at regions of higher contact. A contact value of 1 would represent contact with the ligand for the entire simulation. Key residue positions have been highlighted. (d) shows the same as (c), but for Ac_1PIM5 (purple) and PP (blue).

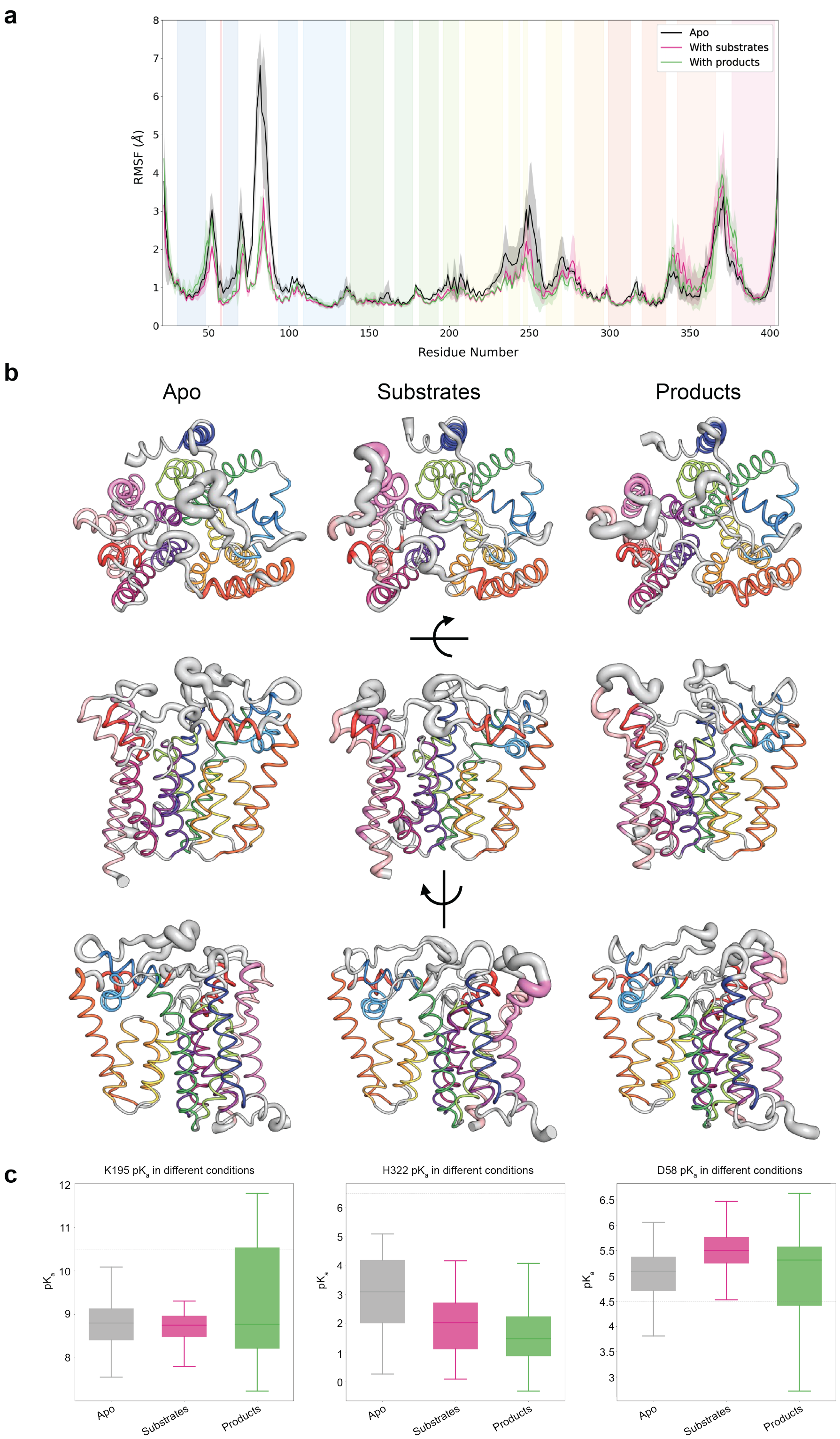

Supplementary Fig. 11 Molecular dynamics analysis of PimE and protonation states.

(a) A plot showing the RMSF for each residue as an average of three independent simulations, with the standard error shown as a faint outline. Simulations without substrates shown in black, those with the substrates shown in pink and simulations with products included shown in green. The different regions of the protein as shown in Fig. 1f are highlighted by color. (b) The RMSF shown as cartoon putty on the PimE structures, where the thicker regions are more mobile in simulations. The helices are colored as in Fig. 1f. (c) pK_a values of selected residues in different simulation conditions. The gray line represents the expected value for that type of residue. The result is shown for each condition as a summary of all three independent repeats with the error bar representing standard error. The first and third quartiles of the data are shown by the box bounds, with the median represented by the line within the box. The whiskers show the furthest data point within 1.5x of the interquartile range of the box, with outliers omitted for clarity.

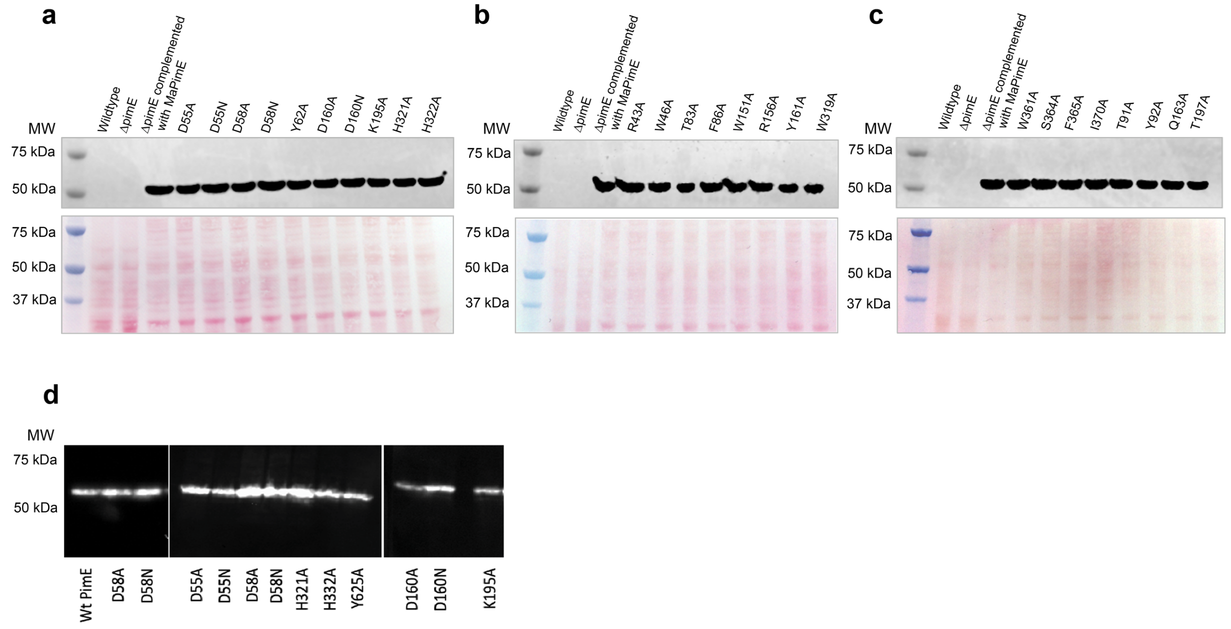

Supplementary Fig. 12 Western blot confirming *Ma*PimE expression levels.

(a-c) Western blot (upper panels) analyses correspond to HPTLC analysis of PIMs profiles shown in (Fig. 4g-h). Blots demonstrate comparable expression levels of WT and mutant *Ma*PimE in complemented *M. smegmatis* strains. The Ponceau S staining (lower panels) demonstrates consistent sample loading across lanes and ensures the quality of transfer for each blot. (d) Western blot analysis of WT *Ma*PimE and the mutant *Ma*PimE constructs used for performing the enzymatic assays presented in Extended Fig. 7d. The blot demonstrates that all mutants have expression levels similar to the WT enzyme, ensuring that the observed differences in enzymatic activity can be attributed to the specific mutations rather than variations in protein expression.

Supplementary Table1. Cryo-EM data collection and modeling statistics for the apo and substrate-bound structures of *Ma*PimE.

	Apo PimE	PimE bound with products
**Data Collection and processing**		
Microscope	FEI Titan Krios-CEC	FEI Titan Krios-NYSBC
Camera	Gatan K3	Gatan K3
Voltage (kV)	300	300
Electron expose (e-/Å2)	58	58
Defocus range (μm)	-1.2 to -2.2	-0.8 to -2.5
Pixel size (Å)	0.87	0.825
Symmetry imposed	C1	C1
Initial particle images (No.)	5,693,488	12,083,984
Final particle (No.)	145,477	56,510
Final Resolution (Å)	3.02	3.46
FSC threshold	0.143	0.143
**Refinement**		
Model composition		
Non-hydrogen atoms	2882	3019
Protein residues	369	384
Ligands	0	2
Waters	0	0
Mean B factor (Å2)		
Protein	35.69	39.79
Ligands		45.96
R.m.s. deviation		
Bond lengths (Å)	0.003	0.003
Bond angles (°) Validation	0.46	0.48
Clashscore	4	8
Rotamers outlier (%)	0	0
Ramachandran plot		
Favored (%)	96	95
Allowed (%)	4	5
Disallowed (%)	0	0

Supplementary Table 2. Oligonucleotide primers used for site-directed mutagenesis of *Ma*PimE

Mutant	Primer Sequences
D58A	Forward: CTCACGCCTCATGGCGCCGATCTGGTCGCCCTCCACGTGTACGTCAGCGGTCCGGCG<br>Reverse: CGCCGGACCGCTGACGTACACGTGGAGGGCGACCAGATCGGCGCCATGAGGCGTGAG
D58N	Forward: CTCACGCCTCATGGCGCCGATCTGGTCAACCTCCACGTGTACGTCAGCGGTCCGGCG<br>Reverse: CGCCGGACCGCTGACGTACACGTGGAGGTTGACCAGATCGGCGCCATGAGGCGTGAG
D55A	Forward: TATCTCACGCCTCATGGCGCCGCTCTGGTCGACCTCCACGTGTACGT<br>Reverse: ACGTACACGTGGAGGTCGACCAGAGCGGCGCCATGAGGCGTGAGATA
D55N	Forward: TATCTCACGCCTCATGGCGCCAATCTGGTCGACCTCCACGTGTACGT<br>Reverse: ACGTACACGTGGAGGTCGACCAGATTGGCGCCATGAGGCGTGAGATA
Y62A	Forward: GATCTGGTCGACCTCCACGTGGCCGTCAGCGGTCCGGCGACGTTG<br>Reverse: CAACGTCGCCGGACCGCTGACGGCCACGTGGAGGTCGACCAGATC
D160A	Forward: TGGACCGAACCGGTGCGGTCCACCCTGGCCTACGGACAGATCAACGTGTTGTTGATG<br>Reverse: CATCAACAACACGTTGATCTGTCCGTAGGCCAGGGTGGACCGCACCGGTTCGGTCCA
D160N	Forward: TGGACCGAACCGGTGCGGTCCACCCTGAACTACGGACAGATCAACGTGTTGTTGATG<br>Reverse: CATCAACAACACGTTGATCTGTCCGTAGTTCAGGGTGGACCGCACCGGTTCGGTCCA
K195A	Forward: ATAGGGCTCGCGGGCGGCGTGGCACTGACCCCACTGGTCAGTGGGCT<br>Reverse: AGCCCACTGACCAGTGGGGTCAGTGCCACGCCGCCCGCGAGCCCTAT
H321A	Forward: CTCATCTCACCGATCTCCTGGACGGCTCACTGGGTGTGGATGGTTCCGT<br>Reverse: ACGGAACCATCCACACCCAGTGAGCCGTCCAGGAGATCGGTGAGATGAG
H322A	Forward: TCACCGATCTCCTGGACGCATGCCTGGGTGTGGATGGTTCCGTTCAT<br>Reverse: ATGAACGGAACCATCCACACCCAGGCATGCGTCCAGGAGATCGGTGA
R43A	Forward: GGGCTGAGTGTCGCAGTGGCCCTGGCGTGGGCATATCTC<br>Reverse: GAGATATGCCCACGCCAGGGCCACTGCGACACTCAGCCC
W46A	Forward: GTCGCAGTGCGCCTGGCGGCGGCATATCTCACGCCTCATG<br>Reverse: CATGAGGCGTGAGATATGCCGCCGCCAGGCGCACTGCGAC

T83A	Forward: GAGTTCACTTACCCGGACAAAGCCCCAGACTTCCCGCTGCCC<br>Reverse: GGGCAGCGGGAAGTCTGGGGCTTTGTCCGGGTAAGTGAACTC
F86A	Forward: CTTACCCGGACAAAACCCCAGACGCCCCGCTGCCCTTTACTTATCCG<br>Reverse: CGGATAAGTAAAGGGCAGCGGGGCGTCTGGGGTTTTGTCCGGGTAAG
W151A	Forward: GTGTGGACGGCGGTCACGATGGCGACCGAACCGGTGCGGTCCAC<br>Reverse: GTGGACCGCACCGGTTCGGTCGCCATCGTGACCGCCGTCCACAC
R156A	Forward: CGATGTGGACCGAACCGGTGGCGTCCACCCTGGACTACGGACAG<br>Reverse: CTGTCCGTAGTCCAGGGTGGACGCCACCGGTTCGGTCCACATCG
Y161A	Forward: GTGCGGTCCACCCTGGACGCCGGACAGATCAACGTGTTG<br>Reverse: CAACACGTTGATCTGTCCGGCGTCCAGGGTGGACCGCAC
W319A	Forward: CTCATCTCACCGATCTCCGCGACGCATCACTGGGTGTGG<br>Reverse: CCACACCCAGTGATGCGTCGCGGAGATCGGTGAGATGAG
W361A	Forward: CTGCTGTTGATTGGAGTGCCCGCGTTGCTCAGCTTCGCCCAACC<br>Reverse: GGTTGGGCGAAGCTGAGCAACGCGGGCACTCCAATCAACAGCAG
S364A	Forward: GGAGTGCCCTGGTTGCTCGCCTTCGCCCAACCTGACATC<br>Reverse: GATGTCAGGTTGGGCGAAGGCGAGCAACCAGGGCACTCC
F365A	Forward: GTGCCCTGGTTGCTCAGCGCCGCCCAACCTGACATCTGG<br>Reverse: CCAGATGTCAGGTTGGGCGGCGCTGAGCAACCAGGGCAC
I370A	Forward: CTCAGCTTCGCCCAACCTGACGCCTGGCGCATCGATCGTCCGTGG<br>Reverse: CCACGGACGATCGATGCGCCAGGCGTCAGGTTGGGCGAAGCTGAG
T91A	Forward: GACTTCCCGCTGCCCTTTGCTTATCCGCCTTTCGCGGCC<br>Reverse: GGCCGCGAAAGGCGGATAAGCAAAGGGCAGCGGGAAGTC
Q163A	Forward: CGGTCCACCCTGGACTACGGAGCGATCAACGTGTTGTTGATG<br>Reverse: CATCAACAACACGTTGATCGCTCCGTAGTCCAGGGTGGACCG
T197A	Forward: GCGGGCGGCGTGAAACTGGCCCCACTGGTCAGTGGGCTC<br>Reverse: GAGCCCACTGACCAGTGGGGCCAGTTTCACGCCGCCCGC

Supplementary References

1. Baldwin, P. R. & Lyumkis, D. Tools for visualizing and analyzing Fourier space sampling in Cryo-EM. *Prog Biophys Mol Biol* **160**, 53–65 (2021).
2. Zi Tan, Y. *et al.* Addressing preferred specimen orientation in single-particle cryo-EMthrough tilting. *Nat Methods* **14**, 793–796 (2017).
3. Punjani, A., Rubinstein, J. L., Fleet, D. J. & Brubaker, M. A. CryoSPARC: Algorithms for rapid unsupervised cryo-EM structure determination. *Nat Methods* **14**, 290–296 (2017).
